# Hall-plot of the phase diagram for Ba(Fe_1−*x*_Co_*x*_)_2_As_2_

**DOI:** 10.1038/srep28390

**Published:** 2016-06-22

**Authors:** Kazumasa Iida, Vadim Grinenko, Fritz Kurth, Ataru Ichinose, Ichiro Tsukada, Eike Ahrens, Aurimas Pukenas, Paul Chekhonin, Werner Skrotzki, Angelika Teresiak, Ruben Hühne, Saicharan Aswartham, Sabine Wurmehl, Ingolf Mönch, Manuela Erbe, Jens Hänisch, Bernhard Holzapfel, Stefan-Ludwig Drechsler, Dmitri V. Efremov

**Affiliations:** 1 Department of Crystalline Materials Science, Graduate School of Engineering, Nagoya University, Furo-cho, Chikusa-ku, Nagoya 464-8603, Japan; 2 IFW Dresden, P.O. Box 270116, 01171 Dresden, Germany; 3 Dresden University of Technology, Faculty for Natural Science and Mathematics, 01062 Dresden, Germany; 4 Central Research Institute of Electric Power Industry, 2-6-1 Nagasaka, Yokosuka, Kanagawa 240-0196, Japan; 5 Karlsruhe Institute of Technology, Institute for Technical Physics, Hermann von Helmholtz-Platz 1, 76344 Eggenstein-Leopoldshafen, Germany

## Abstract

The Hall effect is a powerful tool for investigating carrier type and density. For single-band materials, the Hall coefficient is traditionally expressed simply by 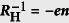, where *e* is the charge of the carrier, and *n* is the concentration. However, it is well known that in the critical region near a quantum phase transition, as it was demonstrated for cuprates and heavy fermions, the Hall coefficient exhibits strong temperature and doping dependencies, which can not be described by such a simple expression, and the interpretation of the Hall coefficient for Fe-based superconductors is also problematic. Here, we investigate thin films of Ba(Fe_1−*x*_Co_*x*_)_2_As_2_ with compressive and tensile in-plane strain in a wide range of Co doping. Such in-plane strain changes the band structure of the compounds, resulting in various shifts of the whole phase diagram as a function of Co doping. We show that the resultant phase diagrams for different strain states can be mapped onto a single phase diagram with the Hall number. This universal plot is attributed to the critical fluctuations in multiband systems near the antiferromagnetic transition, which may suggest a direct link between magnetic and superconducting properties in the BaFe_2_As_2_ system.

It is widely believed that for most unconventional superconductors, Cooper pairs are mediated by spin or orbital fluctuations. A very good example is given by Co-doped BaFe_2_As_2_, one of the most studied Fe-based superconductors (FBS), in which the neutron resonance peak was observed^1^. Note that this resonance peak is hardly elucidated by electron-phonon interaction. Typically, the parent compound of an Fe-based superconductor (FBS) shows a spin-density wave (SDW) phase at low temperatures. This magnetic instability is linked to the Fermi surface (FS) nesting between hole-like pockets centered at the Γ-point and electron-like pockets at M-points in the Brillouin zone^2^. Upon carrier doping, the nesting condition is deteriorated and the superconductivity appears at a given doping level. The emergence of superconductivity in the vicinity of SDW immediately pushed the idea of strong spin fluctuations providing the main glue for Cooper pairing in FBS.

External pressure^3^, chemical pressure^4^, and strain in thin films^5^ may change the nesting conditions similarly to carrier doping. For the latter case, tensile or compressive in-plane strain with biaxial and uniaxial components is induced by the lattice and/or thermal expansion mismatch between film and substrate. Therefore, tensile or compressive in-plane strain may act as control parameter for the phase diagram by selecting a specific substrate.

Here, we report a systematic study of Ba(Fe_1−*x*_Co_*x*_)_2_As_2_ epitaxial thin films grown on MgO(001) and CaF_2_(001) single crystalline substrates by pulsed laser deposition (PLD). The former substrate induces tensile strain, whereas the latter yields compressive one. Using transport data, we construct the phase diagram for Ba(Fe_1−*x*_Co_*x*_)_2_As_2_ thin films under different strain states (i.e., tensile and compressive in-plane strain). The resultant phase diagrams show that the Néel temperature (*T*_N_) and the superconducting transition temperature (*T*_c_) at a given Co doping level depend strongly on the direction of in-plane strain. Both *T*_N_ at zero doping and *T*_c_ at optimal doping level are enhanced by in-plane compressive strain in comparison to single crystals. Moreover, the whole phase diagram is shifted in the direction of higher Co doping. For tensile strain, *T*_N_ at zero doping is reduced and *T*_c_ at optimal doping level is almost unchanged, and the whole phase diagram is shifted to lower doping level. Finally, we demonstrate that the phase diagrams for films on both substrates and single crystals (i.e., the relaxed samples) including magnetic and superconducting regions can be mapped onto a single phase diagram with the Hall number as new variable. Our findings may suggest a direct link between magnetism and superconductivity in FBS.

## Results

### Structural properties

**Figure 1:**
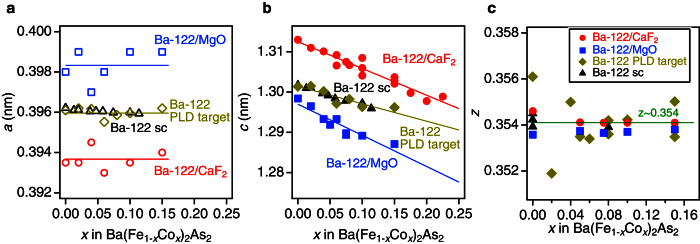
Co doping dependence of lattice parameters. (**a**) In-plane lattice constant *a* of Ba(Fe_1−*x*_Co_*x*_)_2_As_2_ thin films on MgO and CaF_2_ substrates, Ba(Fe_1−*x*_Co_*x*_)_2_As_2_ single crystals (Ba-122 sc)^6^, and PLD target as a function of Co doping. The lines are a guide to the eye. (**b**) The corresponding out-of-plane lattice constants *c* for the same samples. The lines are a guide to the eye. (**c**) The As position (*z*) for the strained films and PLD target materials as a function of the Co doping. The data of bulk single crystals are taken from refs 7, 8, 9, 10. The solid green line shows the average As position for unstrained samples.

All Ba(Fe_1−*x*_Co_*x*_)_2_As_2_ films (0 ≤ *x* ≤ 0.15) were epitaxially grown on MgO(001) and CaF_2_(001) substrates with high phase purity. The epitaxial relation is (001)[100]_film_||(001)[100]_MgO_ and 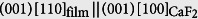. More information on the structural analyses by x-ray diffraction can be found in the Supplementary Information. As shown in Fig. 1a, the lattice constant *a* of the Ba(Fe_1−*x*_Co_*x*_)_2_As_2_ films on CaF_2_ substrates (Ba-122/CaF_2_) is shorter than that of the bulk samples^6^, whereas the opposite relation holds for the Ba(Fe_1−*x*_Co_*x*_)_2_As_2_ films on MgO substrates (Ba-122/MgO). As expected, an elongation of the *c*-axis for Ba-122/CaF_2_ and a shrinkage of the *c*-axis for Ba-122/MgO were observed due to the Poisson effect, as shown in Fig. 1b. These structural changes are due to the biaxial strain with average uniaxial components over sample volume, *ϵ*_*xx*_ = *ϵ*_*yy*_. Here, the average lattice deformations in the tetragonal phase are defined as *ϵ*_*xx*_ = (*a*_film_ − *a*_PLD target_)/*a*_PL Dtarget_ and *ϵ*_*zz*_ = (*c*_film_ − *c*_PLD target_)/*c*_PLD target_ along the *a*- and *c*-axis. In this way, the biaxial in-plane tensile strain acts similarly to uniaxial pressure along the *c*-axis, while the in-plane compressive strain acts as negative pressure along the *c*-axis. The average lattice deformations at room temperature (RT) for Ba-122/MgO along the *a*- and *c*-axis are *ϵ*_*xx*_ = 5.9 × 10^−3^ and *ϵ*_*zz*_ = −4.4 × 10^−3^, respectively. The corresponding values for Ba-122/CaF_2_ films are *ϵ*_*xx*_ = −5.8 × 10^−3^ and *ϵ*_*zz*_ = 5.3 × 10^−3^. It is noted that the lattice deformation for both films are almost constant irrespective of Co contents (Supplementary Fig. S3). The origin of the biaxial strain is discussed in the Supplementary Information.

Figure 1c summarizes the As position (*z*) in the unit cell for the strained films and PLD targets. The literature data for single crystals are also shown in the same figure^7^,^8^,^9^,^10^. It is apparent that the As coordinate is nearly independent of strain and Co doping.

### Resistivity and phase diagram

**Figure 2:**
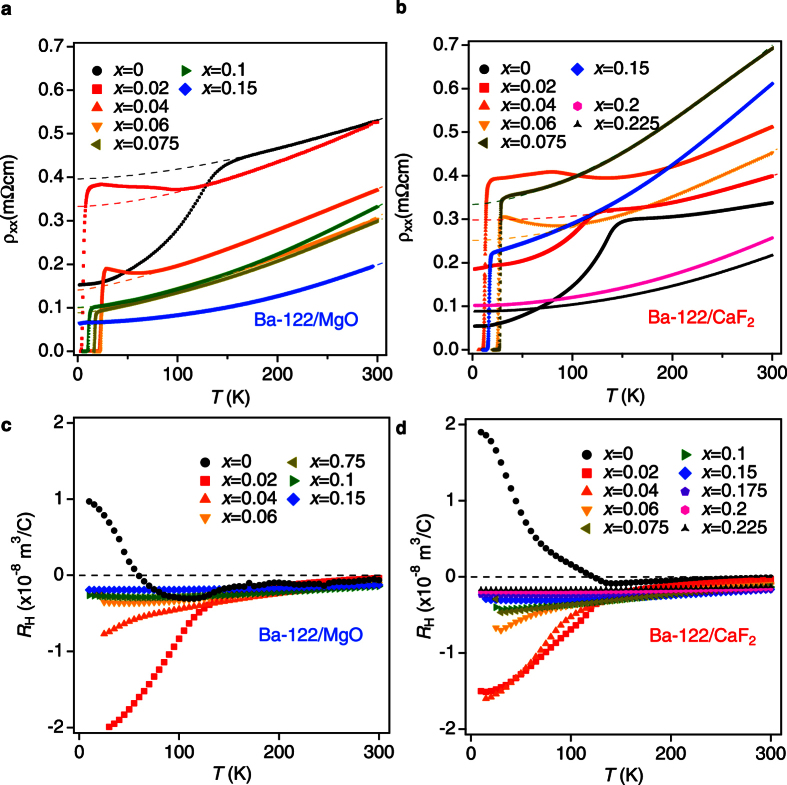
Transport properties of Ba-122/MgO and Ba-122/CaF_2_ thin films. Resistivity data for Ba(Fe_1−*x*_Co_*x*_)_2_As_2_ thin films on (**a**) MgO and (**b**) CaF_2_ substrates. Broken lines are the fitting curves using *ρ* = *ρ*_0_ + *AT*^*n*^ in the paramagnetic (PM) state. Hall coefficient of Ba(Fe_1−*x*_Co_*x*_)_2_As_2_ films on (**c**) MgO and (**d**) CaF_2_ substrates as a function of temperature.

The evolution of the in-plane longitudinal resistivity (*ρ*_*xx*_) curves in zero magnetic field as a function of temperature for the Ba(Fe_1−*x*_Co_*x*_)_2_As_2_ films on MgO and CaF_2_ substrates are displayed in Fig. 2a,b, respectively. The low-temperature state in the films on both substrates changes upon doping similar to the bulk material: from antiferromagnetic to superconducting, followed by metallic state^6^. However, the doping levels at which the phase transitions occur depend on the strain state. For Ba-122/MgO, Co doping of *x* = 0.02 induces superconductivity with a *T*_c_ of 7.5 K. Additionally a sudden drop of the Hall coefficient around 100 K due to the SDW transition was observed (see Fig. 2c). Hence, for Ba-122/MgO with *x* = 0.02 superconductivity coexists with antiferromagnetism. On the other hand, Ba-122/CaF_2_ with the corresponding composition did not show superconductivity down to the lowest temperature available in our experiments (i.e., ∼2 K).

**Figure 3:**
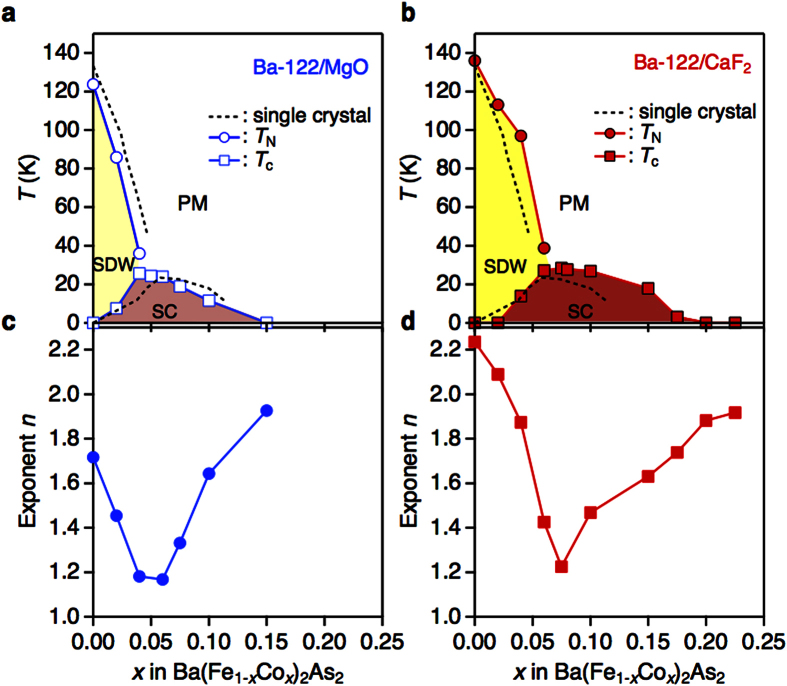
Electronic phase diagram of Ba(Fe_1−*x*_Co_*x*_)_2_As_2_. The electronic phase diagram of thin films grown on (**a**) MgO and (**b**) CaF_2_ substrates. For comparison, the single crystal data^6^,^26^ are also shown in the figures as dotted lines. *T*_N_ and *T*_c_ denote the antiferromagnetic and the superconducting transition temperatures, respectively. SDW, PM, and SC are the spin density wave, paramagnetic, and superconducting phases, respectively. Value for the exponent *n* taken from the resistivity data *ρ* = *ρ*_0_ + *AT*^*n*^ in the paramagnetic state: (**c**) Ba-122/MgO and (**d**) Ba-122/CaF_2_.

Based on the resistivity data, the phase diagrams of both Ba-122/MgO and Ba-122/CaF_2_ are constructed, Fig. 3a,b. For comparison, the single crystal data are plotted in the same figures. Here, *T*_c_ was determined by the superconducting onset temperature (Supplementary Fig. S7), whereas *T*_N_ was defined as a peak position of the temperature derivative of the resistivity curves in analogy to bulk single crystals (Supplementary Information in the section of criterion for *T*_N_)^11^,^12^,^13^. It is noted that the peak position of the temperature derivative of the resistivity is related to the magnetic transition according to x-rays and neutron diffraction measurements^11^. Zero resistivity temperature and middle point of superconducting transition may be influenced by flux pinning effect. Therefore, we chose the onset temperature of resistivity as a criterion of the *T*_c_. It is clear from Fig. 3a that tensile strain (Ba-122/MgO) slightly reduces *T*_N_ and shifts the superconducting dome to lower doping levels compared to the single crystals. A similar shift of the superconducting dome by in-plane tensile strain was observed in P-doped Ba-122 on MgO substrates^14^. To the contrary, biaxial in-plane compressive strain (Ba-122/CaF_2_) effectively pushes the phase diagram to a higher doping level in comparison with single crystals (Fig. 3b). Qualitatively, the shift of the phase diagram can be understood by examining the electronic band structure. At zero doping level, the *ab-initio* calculations show that compressive biaxial in-plane strain makes the band structure more two-dimensional with good nesting (see the section Discussion), resulting in a higher AFM transition temperature. Tensile strain shows the opposite effect which makes the band structure more three-dimensional, and consequently *T*_N_ decreases. For single crystals, a similar development of the FS takes place. Related angle-resolved photoemission spectroscopy (ARPES) measurements showed that upon Co doping the electronic states in the vicinity of the Fermi level become more three-dimensional^15^. Therefore, the two effects (charge doping and in-plane strain) determine the shift of the phase diagram along the doping axis.

The temperature dependence of the resistivity for the films in the paramagnetic (PM) state was fitted using *ρ* = *ρ*_0_ + *AT*^*n*^ and the resultant fitting curves are shown in Fig. 2a,b. This expression has been widely used for analyzing the resistivity in the quantum critical region, e.g. refs 16 and 17. The dependence of the power-law exponent *n* on Co doping (i.e., *x*) is presented in Fig. 3c,d. For Ba-122/MgO, the exponent *n* has a minimum value close to unity at *x* ∼ 0.05. This may be assigned to the AFM quantum critical point (QCP), where the AFM transition temperature goes to zero. For Ba-122/CaF_2_, the QCP is observed at *x* ∼ 0.075 (Fig. 3d). The presence of the AFM QCP for Co-doped Ba-122 has been proposed recently by specific heat, thermal expansion, and nuclear magnetic resonance measurements^18^,^19^,^20^. The observed simultaneous shift of the QCP and the maximum *T*_*c*_ for the strained thin films suggests the relationship between critical magnetic fluctuations and superconductivity in FBS.

**Figure 4:**
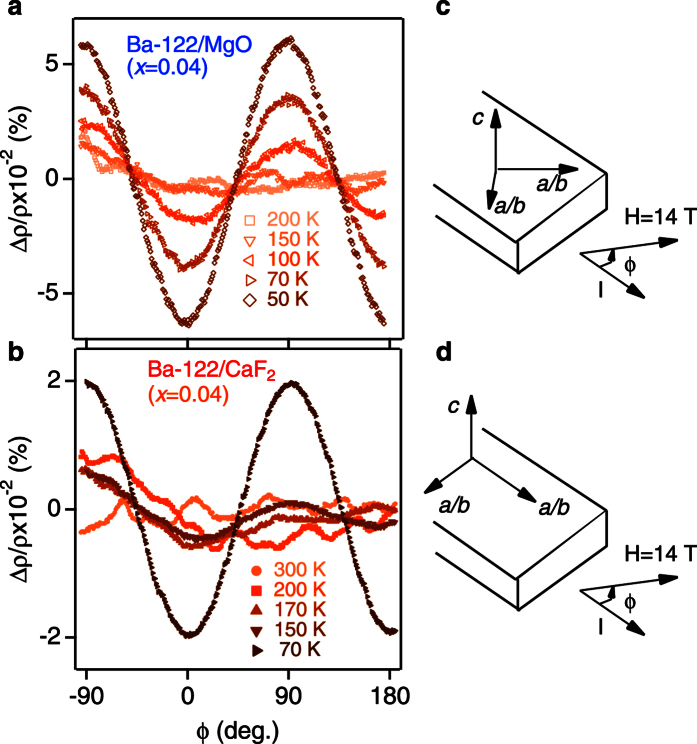
Angular dependence of in-plane magnetoresistance data. The angular dependence of in-plane magnetoresistance (MR) data (Δ*ρ*/*ρ*) in the presence of a magnetic field (14 T) for (**a**) Ba-122/MgO and (**b**) Ba-122/CaF_2_. The sketch gives the orientation of the crystallographic axes for (**c**) Ba-122/MgO and (**d**) Ba-122/CaFv in orthorhombic notations.

In the case of thin films, the substrate may essentially weaken the orthorhombic distortion, as the Ba-122 grains are rigidly fixed at the interface by the substrate. This mechanism is responsible for the strain in the thin films (Supplementary Information). Another evidence for the tetragonal crystal structure being maintained in thin films grown on the substrates comes from the angular dependence of in-plane magnetoresistance (MR) measurements. An example of the in-plane MR for Ba-122/MgO and Ba-122/CaF_2_ with the same Co doping level of *x* = 0.04 in an applied field of 14 T at various temperatures is shown in Fig. 4a,b. As stated above, Ba(Fe_1−*x*_Co_*x*_)_2_As_2_ thin films are grown on MgO(001) substrates with cube-on-cube configuration, whereas the basal plane of Ba(Fe_1−*x*_Co_*x*_)_2_As_2_ is rotated by 45° on CaF_2_(001). We applied the current along the tetragonal [110] direction for Ba-122/CaF_2_ and along the tetragonal [100] direction for Ba-122/MgO, respectively. According to refs 21 and 22, a magnetic field of *B* = 14 T parallel to the *ab*-plane can partially detwin Ba(Fe_1−*x*_Co_*x*_)_2_As_2_ single crystals, leading to a twofold symmetry of the in-plane MR curves below the temperature at which the nematicity sets in. When bias current and magnetic field are parallel to the orthorhombic [100] or [010] axis, the in-plane MR curves show the maximum values. On the other hand, the position of the peak is shifted by 45°, if the bias current (*I*) flows along orthorhombic [110] axis (zero angle corresponds to *B* || *I*. In this case, the peak values are much smaller than for the former geometry (i.e., current and magnetic field || orthorhombic [100] or [010]). However, our results contradict the one obtained from single crystals. Below 100 K, the measured in-plane MR curves for both films clearly follow an almost perfect sinusoidal angle dependence without phase shift. If the oscillation were defined by the nematic domains oriented by the applied field as in the case of single crystals^21^,^22^, the MR signal for Ba-122/MgO should be shifted by 45° with respect to the one for Ba-122/CaF_2_. Additionally, the amplitude of MR signals for both films are quite small compared to those of single crystals. This indicates that the substrate completely blocks the rotation of the nematic/magnetic domains. However, the appearance of oscillation in the MR at a certain temperature *T*^+^ indicates some changes of the FS topology or scattering rates. The *T*^+^ is rather high compared to *T*_N_ and preserved at doping levels above the QCP of the SDW phase. By analogy with refs 23 and 24, *T*^+^ may be related to the nematic phase or fluctuating magnetic domains. This temperature is presumably increased by uniaxial strain if compared to relaxed Ba(Fe_1−*x*_Co_*x*_)_2_As_2_ single crystals.

### Effective carrier density plot of the phase diagram

The temperature dependencies of the Hall coefficients (*R*_H_) measured at 9 T for Ba-122/MgO and Ba-122/CaF_2_ films are shown in Fig. 2c,d. For both parent compound films (i.e., *x* = 0), *R*_H_ is weakly decreasing with decreasing temperature until the SDW transition occurs, similarly to the observation in single crystals^25^,^26^. In contrast to single crystals, however, *R*_H_ changes sign from negative to positive. This behavior can be understood qualitatively by the effect of strain on carrier mobilities. The non-doped Ba-122 is a compensated metal with equal electron and hole carrier densities. Therefore, a small change of the mobilities can strongly affect the experimental value of the effective *n*_H_ (especially in AFM state with reconstructed Fermi surfaces). Only a small amount of Co addition to the system leads to a drastic change in *R*_H_ at low temperature. For both films with *x* = 0.02, *R*_H_ is decreased sharply with decreasing temperature below *T*_N_ due to a large change in the carrier concentration and mobility. This behavior is similar to that observed in single crystals^25^,^26^.

**Figure 5:**
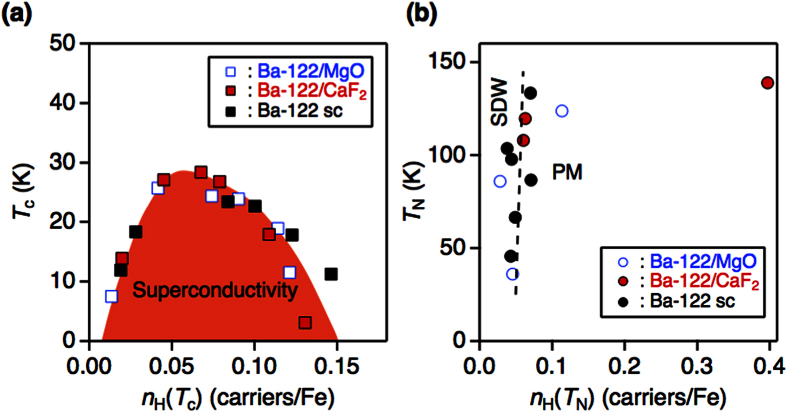
Effective carrier density plot of the superconducting (*T*_c_) and Néel (*T*_N_) temperatures of Ba(Fe_1−*x*_Co_*x*_)_2_As_2_. (**a**) Superconducting (*T*_c_) and (**b**) Néel (*T*_N_) temperatures as a function of *n*_H_(*T*_c_) and *n*_H_(*T*_N_). For comparison, Ba-122 single crystal data taken from ref. 26. are also plotted. SDW and PM are the spin density wave and paramagnetic phases, respectively. The labels show the characteristic range of *n*_H_ in SDW and PM phases.

In order to quantify the effect of strain on the electronic properties, we consider the effective carrier number per Fe, *n*_H_(*T*_c_) and *n*_H_(*T*_N_), as 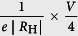, where *V* is the unit cell volume estimated from Fig. 1a,b. Now, we re-plot *T*_c_ and *T*_N_ as a function of *n*_H_(*T*_c_) and *n*_H_(*T*_N_), as shown in Fig. 5a,b. For comparison, the single crystal data from refs 6 and 26 are also shown in the graph. As can be seen, *T*_c_ for both strained thin films and single crystals can be mapped onto a master curve by *n*_H_(*T*_c_) as a new variable. Note that *n*_H_ scales both the position of the superconducting dome and the absolute value of *T*_c_, whereas the carriers numbers^27^ and structural parameters (i.e., bonding angle and anion height)^28^ scale only either the position of the superconducting dome or the absolute value of *T*_c_.

On the other hand, *T*_N_ is vaguely independent of *n*_H_(*T*_N_) for non-zero doping, i.e., a magnetic transition occurs, when *n*_H_(*T*) approaches about 0.05 carriers/Fe.

## Discussion

The shift of the superconducting dome and the AFM transition temperature with uniform in-plane biaxial strain can be understood qualitatively by considering the effect of the strain on the FS shape, its orbital weight, and composition. First of all, our local (spin) density approximation (L(S)DA) calculation shows that the strain and the Co doping affect mainly the hole FS pockets located at the zone center, whereas the electron pockets at the zone corners are nearly unchanged. These results are consistent with the ARPES measurements for doped and undoped Ba-122 single crystals reported recently^29^. Additionally, it was observed that an L(S)DA and generalized gradient approximation (GGA) calculation gives a reasonable prediction of the effect of the Co doping on the band structure^29^. This observation strengthens our theoretical approach.

**Figure 6:**
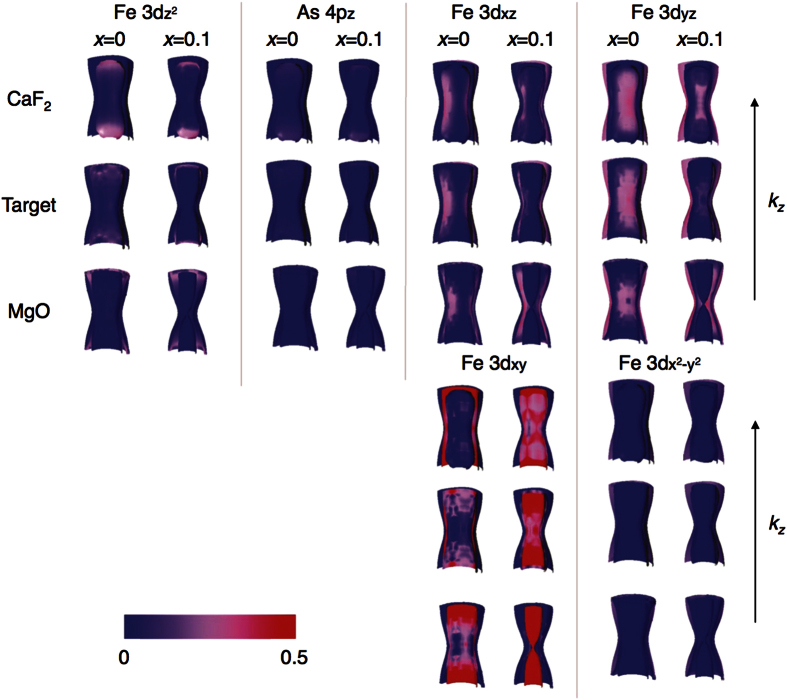
Fermi surface of Ba(Fe_1−*x*_Co_*x*_)_2_As_2_. Evolution of the Fermi surface (FS) of Ba(Fe_1−*x*_Co_*x*_)_2_As_2_ at the Γ point as a function of Co doping and strain. The color code corresponds to a relative orbital weight per Fe-atom. The detailed theoretical approach can be found in Methods section.

Analyzing the changes of the hole FS pockets, we found that *T*_N_ correlates well with the value of the *k*_*z*_ dispersion of the Fe 3*d xz*/*yz* orbitals on the hole FS pockets. As can be seen in Fig. 6, the dispersion along *k*_*z*_ of the undoped Ba-122 film on MgO increases compared to that of the undoped bulk sample (i.e., relaxed) due to tensile strain. *T*_N_ of the former is lower than that of the latter, as shown in Fig. 3a. Simultaneously, the shape of the corresponding FS sheets is getting more three-dimensional. The same trend is observed for different Co doping but with fixed strain state. In contrast, compressive strain alone (i.e., Ba-122/CaF_2_ with fixed Co doping) reduces the *k*_*z*_ dispersion of the Fe 3*d xz*/*yz* orbitals, which leads to the enhancement of *T*_N_. The observed three-dimensional effects of the FS are responsible for the suppression of the FS nesting conditions found by the ARPES study^29^. One can also see from Fig. 3a,b that the degree of shift in the superconducting dome along the doping axis correlates well with *T*_N_; lower/higher *T*_N_ pushes the SC dome towards the underdoped/overdoped region. Such a tendency may be understood if Cooper pairing and magnetic ordering is controlled by a common parameter.

The result of the band structure calculation is insufficient for the interpretation of the observed behavior shown in Fig. 5b. The scaling indicates that the value of the effective carrier number *n*_H_(*T*_N_) at the phase transition is not only related to the electronic structure but also strongly affected by critical fluctuations which are not included in the band structure calculations. As was shown by Kontani *et al*., *n*_H_ scales with the antiferromagnetic (AF) correlation length (ξ^−2^) in the case of strong AF spin fluctuations^30^. Therefore, the value of *n*_H_ should approach zero at the phase transition. However, *n*_H_(*T*_N_) tends to a finite value of ∼0.05 carriers/Fe (excluding films with zero doping level) at the magnetic transition as can be seen in Fig. 5b. This seeming contradiction may be explained by considering the multiband nature of the FS. The Hall coefficient is defined as *R*_H_ = *σ*_*xy*_/*H*(*σ*_*xx*_*σ*_*yy*_), where *σ*_*xy*_ and *σ*_*xx*(*yy*)_ are the full conductivities summed up over all bands. Therefore, the Hall number is given by 
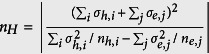
, where *σ*_*h*(*e*),*i*(*j*)_ is the conductivity of a hole (h) or an electron (e) band, *i* or *j* is the summation index running over different hole or electron bands, respectively, and *n*_*h*(e)_ is the corresponding carrier density^31^. Hence, as in the case of resistivity^32^, the bands with the largest conductivities contribute most to the Hall number. At the phase transition, the conductivities of the interacting bands or part of the bands tend to zero due to strong scattering of their quasi-particles on the critical fluctuations. Therefore, *n*_H_ is simply determined by those parts of the FS which are less sensitive to critical fluctuations. This explains why the magnetic transition occurs at a finite *n*_H_ value. Good scaling for *T*_c_ is observed for each side of the superconducting dome, since those regions are far from the magnetic transition lines. In this case, the value of *n*_H_(*T*_c_) is sensitive to the distance to the SDW line. Therefore, the scaling for *T*_c_ can be interpreted by the indication of a strong interplay between *T*_c_ and the magnetic fluctuations slightly above *T*_c_, which are sensitive to the carrier doping and strain as well. A possible disorder effect on both *T*_c_ and *T*_N_ cannot be separated from the spin fluctuation effect, since impurity scattering is also included in *n*_H_.

In conclusion, we have shown that strain essentially affects the phase diagram of the generic system Ba(Fe_1−*x*_Co_*x*_)_2_As_2_. The biaxial in-plane strain is responsible for a nearly rigid shift of the whole phase diagram including the magnetic and superconducting regions along the electron doping. This behavior is explained by band structure calculations in which biaxial in-plane strain affects the FS similar to Co doping. Moreover, the superconducting dome is rigidly connected to the position of the SDW line. The direct relationship between the paramagnetic normal state and *T*_N_, as well as the relationship between *T*_c_ and the preceding state above *T*_c_ are given by the unusual plot of *T*_N_ and *T*_c_ with the Hall number at those temperatures. This emphasizes a crucial role of the critical fluctuations for superconductivity and magnetism in FBS. It is important to check whether a similar plot exists for other families of the FBS, too. Also, a microscopic explanation of the observed unusual behavior is still lacking. We believe that our experimental results will stimulate future theoretical investigations.

## Methods

### Ba(Fe_1−*x*_Co_*x*_)_2_As_2_ films on MgO(001) and CaF_2_(001) substrates

Ba(Fe_1−*x*_Co_*x*_)_2_As_2_ films of around 100 nm thickness have been grown on MgO(001) and CaF_2_(001) substrates by pulsed laser deposition (PLD). PLD targets made by a solid state reaction with various Co levels ranging from 0 ≤ *x* ≤ 0.225 were ablated by a KrF excimer laser with a laser repetition rate of 7 Hz. Prior to the deposition, the substrates were heated to 850 °C. A base pressure of around 10^−8^ mbar at 850 °C was achieved, which increased to 10^−7 ^mbar during the deposition. The thicknesses of all films (∼100 nm) were measured by scanning electron microscope images of cross-sectional focused ion beam (FIB) cuts. In a previous investigation on Ba(Fe_0.92_Co_0.08_)_2_As_2_ films (nominal composition *x* = 0.08) by energy dispersive X-ray spectroscopy, we determined the Co content to 0.74 ± 0.017, indicating good agreement with the nominal value^33^.

### Structural analyses by x-ray diffraction

The *c*-axis texture and phase purity were investigated by x-ray diffraction in Bragg-Brentano geometry with Co-K*α* radiation. In-plane orientation of Ba-122/MgO and Ba-122/CaF_2_ was investigated by using the 103 pole in a texture goniometer operating with Cu-K*α* radiation. In order to precisely evaluate the lattice parameter *a* of Ba-122/MgO and Ba-122/CaF_2_, high resolution reciprocal space maps (RSM) around the 109 reflection were performed with Cu-K*α* radiation.

Temperature evolution of the lattice constants *c* for Ba-122/MgO and Ba-122/CaF_2_ was investigated by x-ray diffraction in Bragg-Brentano geometry with Cu-K*α* radiation in flowing He gas atmosphere. Diffraction patterns were acquired at elevated temperatures from 298 K to 773 K (Supplementary Fig. S4).

### Determination of As position (*z*)

The As position of the PLD target materials was refined by Rietveld analysis using powder x-ray data. For the thin films, *z* was calculated by using the experimental lattice constants *a* and *c*, shown in Fig. 1a,b, and the optimized As position in the paramagnetic state, which is the same method as described in ref. 32.

### Transmission electron microscopy (TEM)

The samples for TEM analysis were prepared using a FIB (SMI3050MS2) by cutting and milling the identical films used for transport measurements. The microstructure near the interface of Ba-122/MgO with *x* = 0.06 was analyzed using JEOL JEM-2100F (Supplementary Fig. S5b).

### In-plane transport measurements

Prior to the micro bridge fabrication, the temperature dependence of the resistance for all films was measured by a 4-probe method, in which small pins are aligned co-linear on the film surfaces. After the measurements, the films were photolithographically patterned and ion-beam-etched to fabricate a small bridge of 100 *μ*m width and 0.41 mm length for transport measurements. No changes in transport properties after the micro bridge fabrication have been found. Longitudinal and transverse resistance were measured with four-probe configuration by a Quantum Design physical property measurement system (PPMS) up to 14 T.

### Theoretical analysis

To understand the impact of the strain on the electron band structure, we performed density functional theory (DFT) calculations of the Fermi surface (FS) for both Ba(Fe_1−*x*_Co_*x*_)_2_As_2_ thin films and bulk single crystals. Our calculations were carried out within the local (spin) density approximation (L(S)DA) using the Full Potential Local Orbital band structure package (FPLO, http://www.fplo.de)^34^. The Co doping was taken into account within the virtual crystal approximation. As can be seen in Fig. 1c, *z* is almost constant irrespective of both strain and Co doping. Therefore, the absolute As position is just proportional to the lattice parameters. A *k*-mesh of 12 × 12 × 6 *k*-points in the whole Brillouin zone was employed. The calculations were performed using the FPLO Ab-Initio Simulation Package within the Perdew, Burke and Ernzerhof (PBE) functional for the exchange-correlation potential. The calculated hole FS for the Co doping level *x* = 0 and 0.1 are summarized in Fig. 6.

## Additional Information

**How to cite this article**: Iida, K. *et al*. Hall-plot of the phase diagram for Ba(Fe_1–*x*_Co_x_)_2_As_2_. *Sci. Rep.* **6**, 28390; doi: 10.1038/srep28390 (2016).

## Supplementary Material

Supplementary Information
