# Supplementary material for: Hall-plot of the phase diagram for Ba(Fe1−xCox)2As2
Source: Sci Rep. 2016 Jun 22;6:28390. doi: 10.1038/srep28390 (PMC4916655; doi:10.1038/srep28390)
Supplement: Supplementary Information [file srep28390-s1.pdf]

Supplementary Information

Hall-plot of the phase diagram for $Ba(Fe_{1-x}Co_x)_2As_2$

Kazumasa Iida,[1,2,*] Vadim Grinenko,[1,2] Fritz Kurth,[2,3] Ataru Ichinose,[4] Ichiro Tsukada,[4] Eike Ahrens,[2,5] Aurimas Pukenas,[5] Paul Chekhonin,[5] Werner Skrotzki,[5] Angelika Teresiak,[2] Ruben Hühne,[2] Saicharan Aswartham,[2] Sabine Wurmehl,[2,5] Ingolf Mönch,[6] Manuela Erbe,[2,7] Jens Hänisch,[2,7] Bernhard Holzapfel,[7] Stefan-Ludwig Drechsler,[2] and Dmitri V. Efremov[2]

[1]*Department of Crystalline Materials Science Graduate School of Engineering, Nagoya University, Furo-cho, Chikusa-ku, Nagoya 464-8603, Japan*
[2]*IFW Dresden, P.O. Box 270116, 01171 Dresden, Germany*
[3]*Dresden University of Technology, Faculty for Natural Science and Mathematics, 01062 Dresden, Germany*
[4]*Central Research Institute of Electric Power Industry, 2-6-1 Nagasaka, Yokosuka, Kanagawa 240-0196, Japan*
[5]*Dresden University of Technology, 01062 Dresden, Germany*
[6]*IFW Dresden, 01171 Dresden, Germany*
[7]*Karlsruhe Institute of Technology, Institute for Technical Physics, Hermann von Helmholtz-Platz 1, D-76344 Eggenstein-Leopoldshafen, Germany*

(Dated: May 12, 2016)

Structural characterization by x-ray diffraction

Our $Ba(Fe_{1-x}Co_x)_2As_2$ (Ba-122) thin films on MgO(001) and CaF_2(001) substrates have been grown by pulsed laser deposition (PLD). The composition of the films is almost identical to that of the $Ba(Fe_{1-x}Co_x)_2As_2$ PLD targets, indicative of a successful stoichiometric transfer [S1]. X-ray diffraction patterns for $Ba(Fe_{1-x}Co_x)_2As_2$ thin films on MgO and CaF_2 substrates as a function of the Co content are summarized in Fig. S1. Almost all peaks are assigned as $00l$ reflections of $Ba(Fe_{1-x}Co_x)_2As_2$ and substrate, indicating a c-axis texture of $Ba(Fe_{1-x}Co_x)_2As_2$. For both cases, the 002 reflection of Fe is observed. The 008 peak position shifts towards higher angles with Co doping (Figs. S1b and S1d), as a result of a smaller lattice constant c with increasing Co content.

ϕ scans of the 103 peak for Ba-122 films on both MgO and CaF_2 are summarized in Figs. S2a and S2b, respectively. For both films, ϕ scans of the 220 substrate reflection were also measured. Sharp and strong reflections from Ba-122 are observed at every 90°. These results highlight that $Ba(Fe_{1-x}Co_x)_2As_2$ thin films are grown epitaxially. Here, the respective epitaxial relation for Ba-122 on MgO and CaF_2 substrates are $(001)[100]_{\rm film}\|(001)[100]_{\rm MgO}$ and $(001)[110]_{\rm film}\|(001)[100]_{\rm CaF_2}$. The films on CaF_2 (Ba-122/CaF_2) with high doping regime ($x \geq 0.175$) contained a small amount of in-plane 45 ° rotated domains.

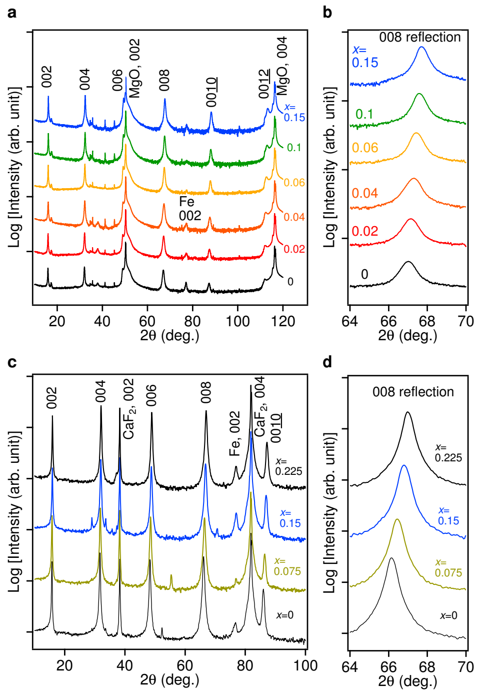

FIG. S1. The $\theta - 2\theta$ scan of the $Ba(Fe_{1-x}Co_x)_2As_2$ thin films on (a) MgO(001) and (c) CaF_2(001) substrates. The $\theta - 2\theta$ scans in the vicinity of the 008 reflection for (b) MgO(001) and (d) CaF_2(001) substrates, respectively. A clear shift of the diffraction peak is observed with increasing Co content.

Ba-122/CaF_2 shows smaller full-width at half-maximum values of both out-of-plane and in-plane reflections compared to Ba-122/MgO, as shown in Figs. S2c and S2d. Here, the rocking curve of the 004 reflection was measured for all Ba-122 films. The $\Delta\omega$ and average $\Delta\phi$ values are mainly constant regardless of Co content.

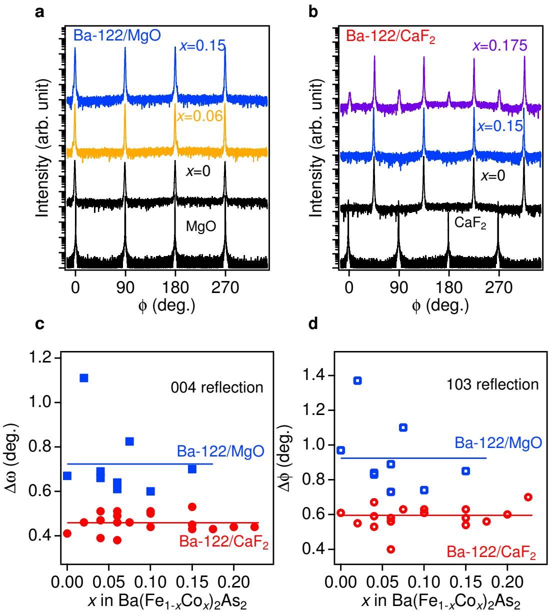

FIG. S2. 103 ϕ-scans for Ba-122 thin films with different Co levels on (a) MgO and (b) CaF_2 single crystalline substrates. The 220 ϕ-scans for MgO and CaF_2 substrates are also shown in the same graphs. (c) Full width at half maximum ($\Delta\omega$) value of the 004 reflection of Ba-122 thin films as a function Co content prepared on various substrates. (d) Average full width at half maximum ($\Delta\phi$) value of the 103 reflection of Ba-122 thin films as a function Co content prepared on various substrates.

Lattice deformation in a tetragonal phase for both Ba-122/MgO and Ba-122/CaF_2 is summarized in Figs. S3a and S3b. Clearly, the lattice deformation for both films are almost constant regardless of Co content.

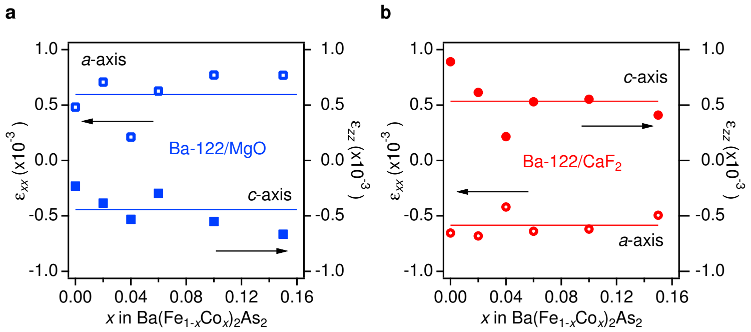

FIG. S3. The lattice deformation in a tetragonal phase along the crystallographic a- and c-axis for Ba-122 thin films on (a) MgO and (b) CaF_2 substrates.

Origin of biaxial strain

The origin of the biaxial strain in thin films may be understood by considering the evolution of the lattice constants c with temperature. The temperature dependence of x-ray diffraction patterns in the vicinity of the 004 reflection of Co-doped Ba-122 films with the same Co doping, $x = 0.15$, but grown on different substrates MgO and CaF_2, are presented in Figs. S4a and S4b. Our measurements have been conducted in flowing He gas. For both Ba-122 films, the diffraction peaks are shifted toward lower angle, indicative of the elongation of the lattice constant c due to thermal expansion. For Ba-122/MgO, the diffraction intensity is observed to decrease around 673 K and almost disappeared at 773 K, indicating that the Ba-122 phase was decomposed. On the other hand, for Ba-122/CaF_2 films the diffraction peak was still observed even at 773 K, although the peak height is significantly reduced. From the temperature dependent x-ray measurements, the lattice parameters a of MgO and CaF_2 substrates have also been evaluated, as shown in Fig. S4c. The evaluated values of the linear thermal expansion coefficients, α, are given in Table S1. Based on those results, the temperature dependencies of the lattice constants c for the two films are presented in Fig. S5a. For comparison, the temperature dependence of the c-axis length of a Ba-122 single crystal is also shown. These data were calculated using the experimental lattice constant at room temperature and the thermal expansion coefficient for the single crystal with $x = 0.115$ [S2]. The lattice constants c for the Ba-122/CaF_2 and the single crystal are close to each other at high temperature, indicating that the respective in-plane lattice parameters a are also close to each other at that temperature. Upon cooling, the difference in the c-axis length between the Ba-122/CaF_2 and the single crystal increases, which is attributed to a large difference in the thermal expansion coefficients of the CaF_2 substrate and Ba-122 (Table S1). This effect is mainly responsible for the compressive strain of $\epsilon_{xx} = -5.8 \times 10^{-3}$ in Ba-122/CaF_2 thin films.

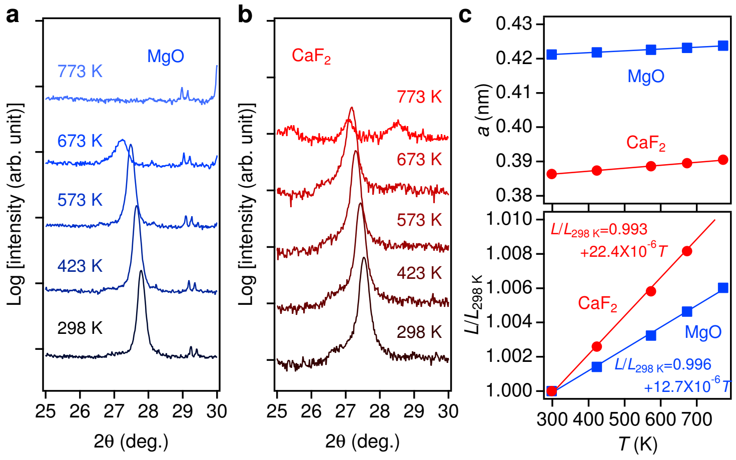

FIG. S4. Temperature dependence of the x-ray diffraction patterns in the vicinity of the 004 reflection for (a) Ba-122/MgO and (b) Ba-122/CaF_2. (c) Temperature dependence of the lattice constants a for MgO and CaF_2 substrates and the corresponding normalized values at 298 K.

On the other hand, the two traces of the Ba-122/MgO film and the single crystal are almost parallel, presumably due to a small thermal expansion mismatch (Table S1). Unlike Ba-122/CaF_2 films [S3, S4], a clean interface between the Co-doped Ba-122 film and the MgO substrate has been observed by transmission electron microscopy, as shown in Fig. S2b and reported in Ref. [S5]. In this case, the lattice misfit yields a tensile strain in the thin films. However, the measured magnitude of the strain, $\epsilon_{xx} = 5.9 \times 10^{-3}$, is smaller than expected for the relatively large lattice misfit of around -6 % at room temperature. This

TABLE S1. The linear thermal expansion coefficient, α, of the MgO and CaF_2 substrates at 298 K. The value for $Ba(Fe_{0.885}Co_{0.115})_2As_2$ along the crystallographic a-axis at 300 K was taken from Ref. S2.

	MgO	CaF_2	$Ba(Fe_{0.885}Co_{0.115})_2As_2$
$\alpha\,(\times 10^{-6}\,K^{-1})$	12.4	22.4	8.5

larger difference is caused by strain relaxation since our Ba-122 films have a thickness of about 100 nm, which is beyond the critical thickness for relaxation. Indeed, our previous investigations on the Ba-122/Fe bilayer system revealed a critical thickness of around 30 nm for a lattice misfit of -2.5% [S6], whereas the corresponding value of Ba-122/MgO results in a few atomic layers [S5]. Therefore, the presence of a small amount of biaxial strain, $\epsilon_{xx} = \epsilon_{yy} = 5.9 \times 10^{-3}$, indicates that residual strain exists beyond the critical thickness, which was also observed in III-V semiconductors [S7] and P-doped Ba-122 films on MgO substrates [S8, S9]. Additionally, we have found nanoscale oscillation of uniaxial strain components using high resolution electron backscatter diffraction (HR-EBSD) [S10], which will be discussed later. This strain state can be described by the modulation of ϵ_{xx} and ϵ_{yy} having opposite sign in the range of about $\pm 0.2\%$ with $\epsilon_{zz} \sim 0$. We assume that this strain component originates from the formation of a low-angle grain boundary network during the coalescence of slightly rotated nanoscale islands nucleating on the mismatched MgO surface during film growth. These strain inhomogeneities were detected by HR-EBSD using a comparable experimental and evaluation procedure provided in reference [S11].

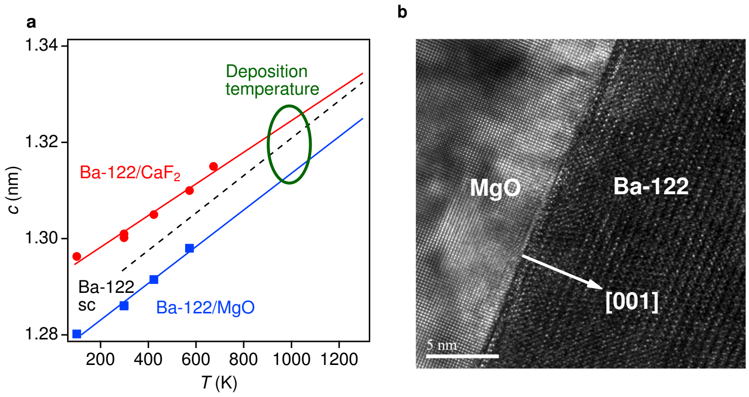

FIG. S5. Temperature dependence of the out-of-plane lattice constants and microstructure: (a) Temperature dependence of the lattice constants c for a Ba-122/MgO and a Ba-122/CaF_2 film with $x = 0.15$. The single crystal data (dotted lines) were estimated from Ref. [S2]. (b) TEM picture in the vicinity of the interface between the Co-doped Ba-122 film ($x = 0.06$) and the MgO substrate.

The local strain by HR-EBSD

Local strain components in a Co-doped Ba-122 ($x = 0.06$) thin film on MgO were measured by high resolution electron backscatter diffraction (HR-EBSD). HR-EBSD was performed as line scans in a Zeiss Ultra 55 scanning electron microscope using 20 kV acceleration voltage and 10 nm step size. The EBSD patterns have been recorded and analyzed subsequently with an in-house written software, based on the algorithm developed by Wilkinson *et al* [S10]. Shown in Fig. S6 is the local strain distribution of the

crystallographic a, b, and c direction (normal strains) relative to a chosen reference position as a function of position. Variations of strain for all directions are within $\pm 0.2\%$.

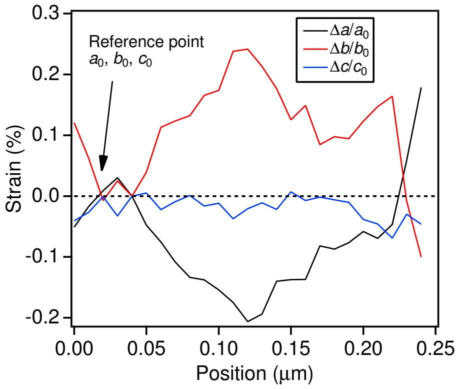

FIG. S6. The local strain distribution of the crystallographic a, b, and c direction as a function of position.

Determining the superconducting transition temperature, T_c

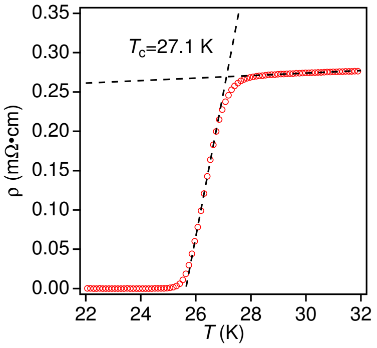

FIG. S7. The resistivity curve of a 10 % Co-doped Ba-122 thin film on a CaF_2 substrate. T_c was determined to 27.1 K.

The superconducting onset transition temperature was defined as the intersection between the linear fit of the normal state resistance and the steepest slope of the superconducting transition (see Fig. S7). Zero resistivity temperature and middle point of superconducting transition are influenced by flux pinning effect. Therefore, we chose the onset temperature of resistivity as a criterion of the T_c. Our optimally Co-doped Ba-122 superconducting films showed an exact match of the zero resistivity temperature $T_{c,0}$ and the onset T_c from magnetization measurements, proving high quality of our films [S1].

Determining the magnetic transition temperature, T_N

The peak position of the temperature derivative of the resistivity is related to the magnetic transition according to x-rays and neutron diffraction measurements [S12]. Therefore, the magnetic transition tem-

perature, T_N, was defined as the peak position, as shown in Fig. S8. In contrast to bulk single crystals (i.e., unstrained material), the kink above T_N related to the structural/nematic transition is absent. Recently, a similar behavior was observed under application of uniaxial strain to the ab-plane of Co-doped Ba-122 single crystals [S13]. Therefore, the uniaxial component in the films obscures the nematic/structural transition (Fig. S6). However, inhomogeneous strain does not affect T_N and T_c noticeably, since the transitions are rather sharp in the strained films.

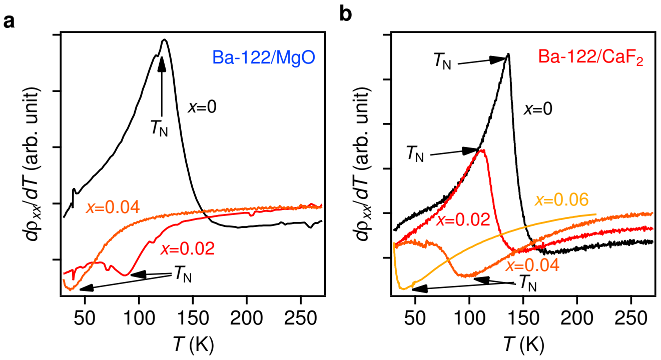

FIG. S8. The temperature derivative of the resistivity curves of (a) Ba-122/MgO and (b) Ba-122/CaF_2. The peak position is assigned as T_N

.

---

* Electronical address: iida@nuap.nagoya-u.ac.jp

[S1] Daghero, D. *et al.* Advanced surface characterization of $Ba(Fe_{0.92}Co_{0.08})_2As_2$ epitaxial thin films. *Appl. Surf. Sci.* **312**, 23-29 (2014).
[S2] da Luz, M. S. *et al.* High-resolution measurements of the thermal expansion of superconducting Co-doped $BaFe_2As_2$. *Phys. Rev. B* **79**, 214505 (2009).
[S3] Kurth, F. *et al.* Versatile fluoride substrates for Fe-based superconducting thin films. *Appl. Phys. Lett.* **102**, 142601 (2014).
[S4] Ichinose, A. *et al.* Induced lattice strain in epitaxial Fe-based superconducting films on CaF_2 substrates: A comparative study of the microstructures of SmFeAs(O,F), $Ba(Fe,Co)_2As_2$ and $FeSe_{0.5}Te_{0.5}$. *Appl. Phys. Lett.* **104**, 122603 (2014).
[S5] Hiramatsu, H. *et al.* Microstructure and transport properties of [001]-tilt bicrystal grain boundaries in iron pnictide superconductor, cobalt-doped $BaFe_2As_2$. *Mat. Sci. Eng. B* **177**, 515 (2012).
[S6] Engelmann, J. *et al.* Strain induced superconductivity in the parent compound $BaFe_2As_2$. *Nat. Commun.* **4**, 2877 (2013).
[S7] Westwood, D. I. & Woolf, D. A. Residual strain measurements in thick $In_xGa_{1-x}As$ layers grown on GaAs (100) by molecular beam epitaxy. *J. Appl. Phys.* **73**, 1187 (1993).
[S8] Kawaguchi, T. *et al.* The strain effect on the superconducting properties of $BaFe_2(As,P)_2$ thin films grown by molecular beam epitaxy. *Supercond. Sci. Technol.* **27**, 065005 (2014).
[S9] Sakagami, A. *et al.* Critical current density and grain boundary property of $BaFe_2(As,P)_2$ thin films. *Physica C* **494**, 181 (2013).
[S10] Wilkinson, A. J., Meaden, J. & Dingley, D. J. High-resolution elastic strain measurements from electron backscatter diffraction patterns: new levels of sensitivity. *Ultramicroscopy* **106**, 307-313 (2006).
[S11] Chekhonin, P. *et al.* Strain inhomogeneities in epitaxial $BaFe_2As_2$ thin films. *Cryst. Res. Technol.* **50**, 891-902 (2015).

[S12] Pratt, D. K. *et al.* Coexistence of Competing Antiferromagnetic and Superconducting Phases in the Underdoped $Ba(Fe_{0.953}Co_{0.047})_2As_2$ Compound Using X-ray and Neutron Scattering Techniques. *Phys. Rev. Lett.* **103**, 087001 (2009).

[S13] Fisher, I.R., Degiorgi, L. & Shen, Z.X. In-plane electronic anisotropy of underdoped '122' Fe-arsenide superconductors revealed by measurements of detwinned single crystals. *Rep. Prog. Phys.* **74**, 124506 (2011).
